# A qualitative study to inform a parental education intervention for unintentional child injury prevention in rural Nepal

**DOI:** 10.3389/fpubh.2026.1761753

**Published:** 2026-01-27

**Authors:** Santosh Bhatta, Julie Mytton, Asmita Ghimire, Lumanti Manandhar, Isabelle Bray, Hamed Zandian, Sunil Kumar Joshi

**Affiliations:** 1 Centre for Public Health and Wellbeing, University of the West of England, Bristol, United Kingdom; 2 Nepal Injury Research Centre, Kathmandu Medical College, Kathmandu, Nepal

**Keywords:** home safety, IEC material, Nepal, parental education, qualitative research

## Abstract

**Introduction:**

Unintentional home injuries are a leading cause of morbidity and mortality among children under five in Nepal, particularly in rural areas. Despite this burden, culturally appropriate community-based prevention strategies remain limited. This study explored community perspectives to inform the design and delivery of a parental education intervention for childhood home injury prevention.

**Methods:**

A qualitative study was conducted in Sunkoshi Rural Municipality, Sindhupalchok District, in December 2024. Seven focus group discussions were held with 56 mothers of preschool-aged children, and 11 key informant interviews were conducted with Female Community Health Volunteers (FCHVs), health-facility in-charges, a school health nurse, and local government officials. Data were analysed thematically using NVivo 14, guided by the Health Belief Model.

**Results:**

Five major themes were identified: (1) Perceived household hazards and common child injuries, (2) Behaviours leading to child injuries, (3) Barriers and facilitators for prevention, (4) Prevention and control practices, and (5) Design and delivery of Information, Education and Communication (IEC) materials. Burns and falls were the most frequently reported injuries, often resulting from unsafe cooking areas, open fires, and poor supervision. Barriers to prevention included limited parental awareness, competing household priorities, and unsafe home environments, whereas community cooperation and FCHV support acted as facilitators. Participants favoured simple, visual, and low-cost educational materials, such as posters, flipcharts, and videos, delivered through participatory group discussions led by FCHVs.

**Conclusion:**

Parents and community stakeholders demonstrated strong interest in home injury prevention education. Embedding culturally tailored parental education within existing community health platforms, particularly FCHVs and mothers’ groups, represents a feasible, scalable, and sustainable approach to reducing childhood injuries in rural Nepal.

## 1 Introduction

Globally, injuries rank as the third leading cause of mortality among children aged 1–4 years, with more than 95% of these deaths occurring in low- and middle-income countries (LMICs) (1). Unintentional home injuries are a leading cause of death and disability among Nepali children under 5 years of age (2, 3). In Nepal, mortality from unintentional injuries among children under five is estimated at 14.93 per 100,000, which is much higher than high-income countries such as the UK at 1.85 per 100,000 (4). This disparity highlights a profound inequality, with children in LMICs bearing a disproportionate burden of preventable injury deaths (5, 6).

Most preschool child injuries in rural Nepal occur at home, particularly in kitchens and courtyards, with falls and burns reported as the most common incidents (7–9). Many of these injuries have multiple preventable components (6, 10), yet they impose potentially major health, social, and economic burdens, including treatment costs and loss of household income due to caregiving (11, 12). However, a culture of fatalism often contributes to the perception that injuries are inevitable, discouraging preventive action (13).

While Nepal has successfully reduced child mortality from communicable diseases due to improved perinatal maternal health, nutrition, and immunisation, injury prevention has not been integrated into child protection or health strategies. The policy documents related to home injuries lack detailed information on injury prevention (9). This gap in integration of injury prevention persists despite Nepal’s ratification of the United Nations Convention on the Rights of the Child (14). Evidence from LMICs demonstrates that parent-focused education programmes can improve knowledge and safety practices (15) and that environmental modifications can reduce household injury risks (16). Yet, Nepal currently lacks culturally appropriate, evidence-based programmes to support parents in promoting home safety.

Given these gaps, there is a clear need to better understand how communities perceive childhood injury risks and what approaches are most acceptable and feasible for delivering preventive education within local contexts. Therefore, this study aimed to explore community perspectives on designing and delivering a culturally and contextually relevant educational intervention for parents to prevent childhood home injuries in Nepal. By providing evidence-based insights, this research seeks to inform the development of the format, content, and delivery methods of an educational intervention that is community-driven, culturally appropriate, and practically implementable through existing community health structures in rural Nepal.

## 2 Methods

### 2.1 Study design and setting

This formative qualitative study comprised focus group discussions (FGDs) with mothers of preschool-aged children and key informant interviews (KIIs) with community stakeholders. The design was chosen to capture parental perspectives, lived experiences, and injury prevention practices, while also integrating system-level insights from local health workers and decision-makers. The Health Belief Model (17) provided the conceptual framework, guiding exploration of perceived risks, benefits, barriers, and cues to action influencing household safety practices.

The study was conducted in Sunkoshi Rural Municipality, Sindhupalchok District, Bagmati Province, Nepal. Sunkoshi covers 72.84 km^2^, comprising seven wards with a population of 15,176 across 4,451 households (18). One ward was purposively selected as the study site following consultation with local authorities and community leaders. It has 2,054 residents in 260 households and includes the communities of Jamune, Kaitar, Dharapani, Tindhare, and Sukekhola. These communities were considered socio-economically diverse, including marginalised families with limited access to child safety information and guidance. This ward is broadly representative of rural hill areas of Nepal, characterised by sloped terrain and mixed livelihoods (18). As hilly regions comprise around 68% of the country, this ward provides a suitable setting for developing a scalable intervention.

### 2.2 Participants

This study was implemented through Nepal’s established community health structures, Female Community Health Volunteers (FCHVs) and mothers’ groups, which serve as trusted platforms for health education (19). FCHVs are trained frontline workers who engage directly with families to promote maternal and child health, while mothers’ groups, coordinated by FCHVs, meet monthly to discuss local health concerns. Two groups of participants included in this study were:

Mothers of preschool-aged children: Mothers with at least one child under 5 years of age, residing in selected ward, were invited to participate in FGDs. Mothers were targeted because of their primary caregiving role and direct influence on child safety practices.

Community stakeholders: KII participants comprised FCHVs, an area health lead, a healthcare manager, a school health nurse, and the ward chairperson. They were selected for their expertise in maternal and child health and their potential involvement in implementing or supporting injury prevention strategies.

### 2.3 Sampling, recruitment, and sample size

Purposive sampling was used to ensure inclusion of diverse perspectives among both mothers and stakeholders. Mothers were recruited through mothers’ groups and FCHVs, while stakeholders were approached via the municipal office and professional networks. Sample sizes were guided by the principle of information power (20, 21), which emphasises adequacy based on study aims, sample specificity, dialogue quality, richness of data and analytic approach rather than statistical representativeness.

Seven FGDs were conducted, each with eight mothers (n = 56). The number of FGDs was chosen to cover the main communities within selected ward, thereby capturing socio-economic and cultural diversity. A group size of eight participants was considered optimal for generating rich discussion while remaining manageable. In addition, 11 KIIs were conducted with stakeholders representing a broad spectrum of health and governance roles.

### 2.4 Data collection

Two semi-structured guides were developed: one for FGDs and another for KIIs. The FGD guide explored mothers’ perceptions of child injury risks, household safety practices, challenges to prevention, and preferred forms of educational support. The KII guide elicited stakeholder views on the burden of child injuries, gaps in parental knowledge and practices, barriers to community-based interventions, and feasible delivery mechanisms.

Both guides were informed by prior research (13), reviewed by experts and pretested with small groups of mothers and health workers to ensure clarity and cultural appropriateness, after which minor modifications were made. These included adding prompts within each discussion topic, incorporating context-relevant examples, and simplifying the content.

FGDs were conducted in familiar community venues such as meeting halls at health facilities, while KIIs were held in private settings including health facilities and municipal offices. Both FGDs and KIIs were conducted over a one-week period in December 2024, with no more than two FGDs or three KIIs conducted per day. All sessions were facilitated in Nepali by two trained qualitative researchers experienced in community engagement. FGDs and KIIs lasted for 30–60 min. All were audio-recorded with participants’ consent, and field notes were taken to capture contextual information and non-verbal cues.

### 2.5 Data management and analysis

Recordings were transcribed verbatim in Nepali and translated into English by bilingual researchers. The translations were verified by cross-checking the English text against the original Nepali audio recordings by other researchers, and all data were anonymised before analysis. All materials were stored securely on password-protected institutional servers.

Data were analysed thematically using Braun and Clarke’s six-step approach (22). Translated transcripts were imported into NVivo 14 (23) to support systematic coding. Two researchers independently coded two sets of transcripts to develop a coding framework, which was then applied across the dataset. Initial coding categories were informed by constructs from the Health Belief Model, including susceptibility, severity, benefits, barriers, cues to action and self-efficacy, while allowing inductive themes to emerge from the data. Coding discrepancies and differences in theme interpretation were discussed iteratively until consensus was reached, and where agreement could not be achieved, a third senior researcher reviewed the data and provided arbitration (24).

Several strategies were used to ensure the rigour and trustworthiness of the qualitative data. Credibility was strengthened through triangulation of focus group discussions and key informant interviews, independent double coding of transcripts, and member checking during and after data collection. Consistency of analysis was maintained through clear documentation of coding decisions, reflexive notes, and regular discussions within the research team to resolve differences in interpretation (25).

### 2.6 Ethical considerations

Ethical approval was obtained from the Institutional Review Committee of Nepal Health Research Council, Nepal (Registration no: 536_2024), and the University of the West of England (UWE) Research Ethics Committee, UK (Registration no: 13224892). Permission to conduct the study was also obtained by the Sunkoshi Rural Municipality and Ward-1 office.

Participants were provided with written information sheets in Nepali, outlining the study purpose, voluntary participation, and right to withdraw at any stage. Written informed consent was obtained prior to data collection; for participants with limited literacy, the form was read aloud, and a thumbprint was accepted. Confidentiality was maintained through anonymisation of transcripts and secure data storage (UWE OneDrive). Findings are reported to prevent the identification of individuals or communities.

## 3 Findings

Findings are presented in two sections: the characteristics of focus group and key informant participants, and the themes generated from the thematic analysis, presented in five key areas.

### 3.1 Participants

#### 3.1.1 Characteristics of focus group participants

A total of 56 women aged 20–40 years participated, with each focus group consisting of 8 participants. All women had children between 2 months and 5 years old. The majority were from the Janajati community, the largest local ethnic group, with three focus groups composed solely of Janajati participants. Two groups included Chhetri members, while one group each included Brahmin and Dalit participants. Details of participant characteristics are provided in Table 1.

**Table 1:** Characteristics of focus group participants.

Focus groups (FGs)	Description	Age range (children)	Length of session	Location/venue
FG1	Janajati Community	15 months to 4 years	36 min	Paharibasti Community Health Unit
FG2	Janajati Community	9 months to 5 years	41 min	Paharibasti Community Health Unit
FG3	Janajati Community	10 months to 4 years	54 min	Dharapani Community Health Unit
FG4	Janajati and Dalit Community	2 months to 4 years	45 min	Dharapani Community Health Unit
FG5	Janajati and Brahmin Community	6 months to 3 years	45 min	Sunkoshi Basic Hospital
FG6	Janajati and Chhetri Community	8 months to 5 years	40 min	Sunkoshi Basic Hospital
FG7	Janajati and Chhetri Community	11 months to 3 years	45 min	Sunkoshi Basic Hospital

#### 3.1.2 Characteristics of key informant interview participants

The demographic characteristics of 11 interviewees are displayed in Table 2. Their years of service varied widely, ranging from 6 months to 36 years, reflecting a mix of experience.

**Table 2:** Characteristics of key informant interview participants.

KII participants	Designation	Age group (in years)	Length of service	Sex	Length of session
KII 1	Female Community Health Volunteer	50–60	36 years	Female	46 min
KII 2	Auxiliary Health Worker	40–50	3 years	Male	54 min
KII 3	Female Community Health Volunteer	40–50	5 years	Female	40 min
KII 4	Auxiliary Health Worker	20–30	3 years	Female	36 min
KII 5	Health Assistant	30–40	9 years	Female	35 min
KII 6	Female Community Health Volunteer	30–40	0.5 years	Female	41 min
KII 7	School Health Nurse	20–30	3 years	Female	43 min
KII 8	Female Community Health Volunteer	30–40	6 years	Female	49 min
KII 9	Healthcare manager	30–40	3 years	Male	33 min
KII 10	Ward Chairperson	30–40	3 years	Male	60 min
KII 11	Area health lead	30–40	4.5 years	Male	47 min

Participants are listed in the order in which the interviews were conducted.

### 3.2 Themes

A total of five themes were identified from FGDs and KIIs and these are listed in the Table 3 and described in the following sections. Each quotation presented in the findings is attributed to the respective participant’s identification code (e.g., FG1-P1 refers to Participant 1 from Focus Group 1; KII1 denotes the participant from Key Informant Interview 1).

**Table 3:** Themes and sub-themes identified through thematic analysis.

Themes	Sub-themes
Perceived hazards and injuries in the home environment	Identified risks and home safety Common injuries among children High-risk places in the home environment
Behaviour leading to injuries	Inability of children to recognize hazards Parental actions contributing to child injury
Factors hindering and enabling child injury prevention	Potential barriers for child injury prevention Potential facilitators for child injury prevention
Prevention and control practices for child injuries	Potential changes in the home environment Secondary prevention practices by parents
Design and delivery of Information, Education and Communication (IEC) materials	Potential IEC materials and content Delivery of IEC materials

#### 3.2.1 Perceived hazards and injuries in the home environment

Participants across all focus groups and interviews described a range of hazards within and around the home that placed young children at risk of unintentional injury. Although the relative emphasis differed slightly between mothers and key informants, there was strong overall agreement that burns, falls, cuts, and choking were the most common injuries experienced by children under five.

Many mothers identified the kitchen as the most hazardous area of the home. The use of open, low-level mud stoves, firewood, hot liquids, and cooking utensils at ground level made young children particularly vulnerable. Mothers described the close proximity between cooking and living spaces, typical in rural homes as a major challenge, noting that it was often difficult to keep mobile toddlers away from heat sources. These accounts were echoed by several key informants, who similarly described kitchens as the site of frequent burn injuries among young children.

Several stakeholders elaborated on why rural kitchens pose such risks, emphasising that design, fuel type, and children’s mobility interact to create hazardous conditions. As one health worker explained:

*“In the kitchen, the stoves are made of mud. The fires are lit with firewood; we don't do it upstairs; we do it downstairs. When the children go crawling there, they don't know they'll get burnt, and when they go there and put their hand in the fire, it obviously burns them, and that is extremely dangerous.” (KII8)*

In contrast, some stakeholders placed greater emphasis on outdoor hazards, especially courtyards and porches, which were characterised by uneven surfaces, loose stones, and sloped terrain. These physical features were reported to contribute to regular falls among children, particularly when running or playing unsupervised. One health worker noted:

*“Based on the cases we've seen, most falls occur outside the house rather than inside… houses are built on slopes, which makes falls from houses and uneven areas common.” (KII9)*

Mothers generally agreed with these observations, especially those living on steeper slopes or in homes with elevated entrances. Although mothers of younger infants (less mobile) focused more on indoor burn risks, mothers of older toddlers more often described falls occurring outdoors. No substantial differences were observed across ethnic groups; however, Janajati mothers living on steeper land more frequently highlighted outdoor fall risks.

Structural features of homes further contributed to these risks. Traditional houses often contained open or unfenced staircases, balconies, and windows, while newer concrete homes, although sturdier, were perceived as increasing the severity of injuries due to hard cement floors, particularly when wet during heavy rains. In hilly area, many homes were built on different levels, with elevated entrances and multiple steps or stairways along access paths, increasing the risk of falls from height, particularly among mobile toddlers. Both mothers and stakeholders highlighted that agricultural tools, sharp household items, and small objects were often stored within reach in courtyards or verandas, posing additional risks such as cuts or choking.

#### 3.2.2 Behaviours leading to injuries

Participants discussed how the developmental limitations of children under five, combined with everyday caregiving circumstances, can unintentionally increase the risk of injuries at home. Across most FGDs and KIIs, participants emphasised that children have a limited understanding of risk and are especially vulnerable because of their natural curiosity. Mothers and stakeholders explained that young children often explore their surroundings or imitate others without recognising potential dangers such as climbing trees, touching electrical sockets, playing near fires, handling sharp objects, or teasing animals.

*“Children under five cannot climb trees, but they might try to imitate their siblings. Even on a low branch, they may attempt to climb and balance between two branches, but such attempts could lead to falls.” (KII2)*

Almost all participants agreed that most injuries occur unintentionally and are often linked to limited supervision resulting from competing household and work responsibilities, rather than deliberate neglect. Mothers explained that continuous supervision was difficult while performing essential tasks such as cooking, fetching water, or farming, and that caring for multiple young children further increased supervision challenges and injury risk, particularly among mobile toddlers.

Almost all participants agreed that injuries usually occur unintentionally and are often linked to limited supervision that results from competing household and work responsibilities rather than deliberate neglect. Mothers described the challenges of watching their children continuously while performing essential tasks including cooking, fetching water, or farming. As one mother explained:

*“Injuries happen when we are busy. Even if one adult is home, we have other tasks like cooking or fetching water. That’s when children fall or get hurt. Sometimes, there is no one around.” (FGD3-P2)*

Participants also described situations where unsafe practices, such as leaving hazardous items within reach, may occur unintentionally due to habit, convenience, or a lack of awareness about potential risks. These actions were not viewed as intentional but as part of everyday routines shaped by practical and environmental constraints.

*“For example, they know that chemicals shouldn't be within a child's reach, yet sometimes convenience leads them to store such items unsafely.” (KII11)*

#### 3.2.3 Factors hindering and enabling child injury prevention

##### 3.2.3.1 Barriers to improving home safety

Participants described a wide range of environmental, cultural, and practical challenges that limited their ability to reduce injury risks at home. Several key informants explained that many families were hesitant to modify long-standing construction practices that had been passed down through generations. Traditional designs such as staircases without handrails, unfenced terraces and balconies remained common even when safer options were known. This reluctance to alter familiar structures reflects a broader perception that major changes are difficult to achieve in everyday rural life.

Mothers also reported lacking the skills or confidence to make physical modifications that could improve home safety, such as fencing courtyards, reorganising storage areas or securing hazardous items. One mother highlighted this issue:

*“There are places for storing sharp items, but many families don’t know how to use them properly, even if they have the space.” (FGD4-P3)*

Geographical barriers, including steep slopes, landslides, and poor road infrastructure, further constrained access to building materials and safety resources. These physical limitations made it difficult for families to alter their homes or surroundings to reduce risks.

Participants consistently described the challenge of supervising young children while meeting the demands of rural livelihoods. Mothers explained that they had to balance childcare with the practicalities of rural living, which sometimes limited their attention to children’s activities.

*“Injuries are hard to prevent, especially for children under five, because of the nature of rural life-housework, fieldwork, and tending to livestock.” (KII3)*

A few key informants mentioned alcohol use in some households as an occasional factor that could reduce adults’ attentiveness to children, although this was not commonly raised by mothers. Economic hardship placed its own pressures on families by increasing the need for income-generating work, which sometimes led parents to rely on older siblings or neighbours for childcare.

Participants also described uncertainty about how to recognise and respond to injuries. This was particularly common among first-time mothers, who sometimes felt unsure about the seriousness of an injury or when to seek help. One mother recalled:

*“I didn’t even realise my child was injured. It was my first child, and I had no knowledge. My child kept crying, and I just tried to comfort him without knowing what was wrong.” (FGD1-P6)*

Some injuries were often managed at home using traditional remedies, reflecting common local practice and uncertainty about when professional care was necessary. Both caregivers and health workers highlighted the need for practical first-aid training to strengthen parents’ confidence in responding to injuries and in seeking timely care. As one informant explained:

*“Immediate response to injuries is crucial, but not all parents have the skills. First-aid training would be highly beneficial.” (KII11)*

##### 3.2.3.2 Facilitators to reduce injuries in and around home

Despite challenges, many participants also identified several enabling factors that could support safer home environments. A commonly expressed view shared by mothers and key informants was the need to raise awareness about child safety across entire households rather than focusing solely on mothers. Educating fathers, grandparents, and older siblings was seen as essential for creating consistent safety practices at home.

*“Awareness programs would definitely help. If we can educate the whole family, not just the mothers, it will have a bigger impact.” (KII3)*

Strong community cohesion, particularly within indigenous Janajati communities, emerged as another facilitator. Mothers described shared norms of collective caregiving in which neighbours and relatives routinely watch out for one another’s children. This communal vigilance was viewed as a natural buffer when caregivers were occupied with daily chores.

*“It’s not a problem because there are other houses nearby, and the courtyard is big. If a child moves too far, there are people to watch over them.” (FGD4-P2)*

Such social connectedness may amplify the effects of injury prevention interventions, as messages delivered to mothers could be reinforced by the wider community. At the same time, a few participants noted that shared responsibility sometimes risks diffusion of responsibility, highlighting the need for clear guidance on supervision roles.

Support from family members, neighbours, and other community members was also emphasised as a valuable resource for both supervision and environmental improvements.

*“When we are building a fence or boundary wall, other family members, neighbours and community members can help by carrying stones, mud, or bamboo. If we are busy with other tasks, they can help by watching over the children." (FGD1-P2)*

Access to nearby health facilities was identified as an enabler, particularly where reliable access routes and functional river bridges reduced travel time despite rugged terrain. Opportunities for skill-based training, including first aid, income-generating activities, and time management, were also viewed as enhancing parents’ ability to create safer environments for their children.

*“Instead of direct financial assistance, they [mothers] should be provided with skill-based training or help in marketing their products to generate income. This approach can be more sustainable and effective.” (KII8).*

#### 3.2.4 Prevention and control practices for child injuries

Participants described a range of practical strategies, both behavioural and environmental, that they believed could help prevent injuries among young children in the home. These suggestions reflected their recognition of children’s high susceptibility to injuries and the potentially severe consequences of everyday hazards, aligning with key constructs of the Health Belief Model.

Across almost all focus groups, mothers emphasised the importance of keeping hazardous items, such as sharp tools, medicines, chemicals, hot liquids, and fire sources, beyond children’s reach. Many mothers explained that such measures required continuous awareness and habitual organisation within the household. As one key informant summarised,

*“The first step is changing our mindset. We need to remind ourselves. There are children in the house, so I should not leave anything within their reach that could cause harm.” (KII3).*

Participants also suggested specific indoor modifications that could reduce common hazards. These included replacing high beds with lower ones, avoiding placing heavy objects on cupboards next to sleeping areas, covering sharp corners with foam, taping electrical sockets, securing water containers, and fully extinguishing fires after cooking. These suggestions were commonly raised across mothers from different ethnic groups and echoed by health workers. As noted by one participant,

*“Children should not be allowed near the fire, and it’s important to fully extinguish fires with water to eliminate any chance of burns” (KII7).*

Outdoor preventive measures focused largely on reducing fall risks in sloped and uneven surroundings. Many mothers described locally feasible changes, such as constructing bamboo fences to mark the home perimeter and prevent children from venturing towards steep edges. A mother explained,

*“I think we needed to lower the height of the porch because children kept falling from it.” (FGD1-P1)*

*“To ensure safety, children should not be left alone, and the house should be fenced properly.” (FGD2-P6)*

Participants from the Janajati community, where homes are typically clustered, reported that fencing was particularly useful in preventing children from wandering into neighbouring courtyards or animal sheds.

Consistent with earlier themes, participants highlighted supervision as the most critical protective factor, though its feasibility varied depending on workload, number of children, and household structure. Participants consistently stated that injuries often occurred when children were left unattended, even for short duration, while caregivers were engaged in household or agricultural work. As one key informant expressed:

*“A person should look after the child at all times, whether it is their mother or someone else. By doing this, half of the risk is already reduced. They don’t get a chance to fall.” (KII4)*

Although “constant supervision” was often described as ideal, many mothers acknowledged that it was challenging within the practical realities of rural living. In households where supervision was shared among extended family or neighbours, mothers reported feeling more confident that children remained safe.

Participants described varied approaches to managing injuries when they occurred. Minor injuries, such as small cuts or bruises, were commonly treated at home, often with traditional herbal remedies that are widely used and culturally accepted. As one key informant noted,

*“For small injuries, they can be treated by homemade remedies like applying grass [medical herbs]. It is still like that; they squeeze the Crofton weed ‘banmara jhar’ and apply it to the wound.” (KII7)*

More severe injuries, particularly burns, deep wounds, or animal related injuries like dog bites, prompted visits to nearby health posts or hospitals. Mothers’ decisions to seek care depended on visible severity, persistence of pain, or uncertainty about the cause of the injury. This variation illustrates differences in perceived severity and cues to action, important considerations for future interventions.

Importantly, participants highlighted the need to enhance awareness and education on child safety at home, emphasising that preventive strategies should respect local cultural practices and beliefs. Many mothers expressed that prevention should not fall solely on mothers but involve fathers, grandparents, and older siblings. Engaging the whole household was viewed as essential to sustaining behaviour change and reinforcing shared responsibility for children’s safety.

#### 3.2.5 Design and delivery of IEC materials

Participants identified diverse IEC materials, key content areas, and preferred delivery approaches to enhance community engagement and support the prevention of child injuries in the community. Mothers across different ethnic groups, including Janajati and Dalit participants, emphasised the need for visual, practical, and easy-to-understand materials that reflect their household environments and daily routines.

Participants suggested a combination of posters, flipcharts, and audiovisual tools to communicate safety messages. Posters were considered simple, accessible, and effective, particularly for audiences with varying literacy levels. Flipcharts were valued for presenting information in a structured and sequential way, supporting both reading and visual engagement. As one participant explained:

*“If we use a flip chart, mothers who can read will understand by reading, and those who cannot still benefit from the pictures. So, both posters and flip charts would be great.” (KII3).*

Several mothers also recommended incorporating short videos that could be viewed on mobile phones. Younger mothers, who use mobile phone, expressed that videos “make it easier to understand because we can see what to do.” The use of visuals, whether printed or digital, was widely regarded as essential for improving understanding among community members with different literacy skills.

Regarding the content, participants emphasised that IEC materials should include images and brief explanatory text to ensure clarity and inclusivity. Mothers wanted visuals that depicted common household risks and realistic preventive actions. Suggested content included safe play areas, fire safety, using barriers to prevent falls, and safe storage of tools, chemicals, and electrical appliances. A mother explained that pictures “help us see what needs to change in our own houses,” especially when the images resemble local housing structures, kitchens, and courtyards.

Participants also highlighted the importance of addressing hazards related to electrical wiring, poisoning, and exposure to livestock. Mothers, particularly those from Janajati households where livestock often share outdoor spaces with children, stressed the importance of showing how to create safe play areas and keep hazardous items out of reach. They felt that using culturally familiar imagery would make messages clearer and more relevant to families in their communities. One key informant noted:

*“If we create posters, we could show images of potential risks, like children being injured in the kitchen or with tools. We can also show unsafe homes and provide solutions.” (KII3).*

Participants emphasised that the effectiveness of IEC materials depended not only on their design but also on how they were delivered. Mothers across all ethnic groups strongly preferred interactive group discussions over one-way information sessions. They explained that learning together made it easier to ask questions, share experiences, and clarify misunderstandings. As one mother said:

*“If we create posters and show them while explaining, people will understand better. Simply listening might not be enough. People hear things, but they might not fully grasp them.” (FGD2-P3).*

Group-based learning was also viewed as more inclusive and time-efficient, particularly in communities where one-to-one outreach is challenging. Similar perception was found in another mothers’ group discussion, where participants emphasized that collective discussion facilitates shared learning and clarification of ideas.

*“If we discuss in a group, one person might understand even if another does not. We can ask, “what did you understand?” This way, it becomes a bit easier.” (FGD7-P5).*

Participants highlighted the importance of skilled facilitators, such as FCHVs, who possess good communication skills, empathy, and the ability to engage parents effectively. Trainers were expected to explain information clearly, demonstrate safety practices, and provide emotional and practical support to encourage behaviour change.

A combination of posters, flipcharts, and short videos, delivered through participatory group discussions led by trained facilitators, was viewed as the most feasible and effective strategy for supporting mothers in improving their knowledge and skills related to home safety practices. However, participants also acknowledged potential challenges, including limited training capacity and insufficient resources for producing and distributing IEC materials.

## 4 Discussion

Guided by the Health Belief Model, this discussion is structured around the five key themes identified in the results section that influence child injury prevention in rural Nepal.

Caregivers recognised multiple household hazards, particularly in kitchens, courtyards and porches, but often underestimated their severity. This indicated variation in how risks were interpreted. Children’s natural curiosity and limited supervision, shaped by competing domestic responsibilities, frequently led to injuries. These accounts highlighted children’s susceptibility and caregivers’ constrained capacity to respond effectively. Barriers such as poverty, traditional practices and limited safety knowledge further hindered prevention. In contrast, strong community networks, family support and access to health services acted as important facilitators. Participants emphasised practical, low-cost prevention measures, including simple home modifications, fencing and increased supervision, which were viewed as both feasible and beneficial. Caregivers preferred visual and participatory IEC tools, such as posters, flipcharts and videos, delivered through group discussions led by trained facilitators. These approaches were considered effective for improving awareness, reinforcing safety behaviours and sustaining community engagement in injury prevention. Overall, the findings show how caregivers’ perceptions of risk, the anticipated value of preventive actions and the practical barriers they face shape their motivation and capacity to adopt safer behaviours.

### 4.1 Perceived hazards and injuries in the home environment

The study identified several environmental hazards that increase children’s risk of injuries. Caregivers’ descriptions reflected a clear awareness of children’s susceptibility to harm, although mothers and key informants sometimes differed in the hazards they prioritised. The triangulation of perspectives revealed a consistent pattern: the combination of indoor and outdoor environmental hazards, and the physical layout of rural hill homes, created multiple overlapping risks for young children. These conditions increase the likelihood of injuries such as burns, falls, cuts, and choking, aligning with findings from similar settings in rural Nepal (2, 13, 26). Although participants recognised these hazards, most demonstrated poor safety practices and limited ability to maintain safer homes, which is consistent with findings from other studies (27, 28). This suggests gaps in both perceived severity and confidence to manage risks. Caregivers and communities should therefore be aware of potential hazards and prioritise safety in the design and maintenance of indoor and outdoor household spaces (16, 29).

### 4.2 Behaviours leading to childhood injuries

Participants described children as highly susceptible to injury, highlighting how natural behaviours interact with the practical realities of rural caregiving. Limited supervision, competing responsibilities, and routine habits can create circumstances in which unintentional injuries are more likely to occur. Most participants attributed childhood injuries to inadequate supervision, as caregivers were often occupied with household chores, a finding echoed with studies from Uganda and Bangladesh (30, 31). While many home injury risks such as access to sharp tools or household chemicals are common across diverse settings, including high-income countries (32), our study identified additional hazards that are strongly shaped by the rural hill context. These included children’s proximity to open mud stoves, increased risk of falls from terraced land, traditional unfenced staircases and porches, and frequent interactions with domestic animals. These environmental conditions may amplify the consequences of typical child behaviours such as exploration and imitation, especially where caregivers perceive limited ability to control these risks. Children’s curiosity and tendency to mimic adult activities, including cooking or handling tools, further highlight the need to strengthen caregiver education and enhance practical supervision strategies (33). This is particularly important in settings where perceived barriers, such as workload and time constraints, impede consistent preventive action.

### 4.3 Factors hindering and enabling child injury prevention

Many of the challenges described by caregivers reflect substantial barriers to taking preventive action. Financial hardship, limited safety knowledge and rural living conditions were major constraints to home safety improvements, consistent with findings from Nepal and Uganda (29, 34). Traditional construction methods such as houses built with unfenced balconies, open staircases, and narrow verandas, typically do not incorporate modern safety features like railings, barriers, or child-safe storage spaces. As a result, families have limited opportunities to integrate protective modifications without undertaking substantial structural changes that are often unaffordable. The sloped terrain and dispersed settlements make transporting building materials difficult and costly, limiting families’ access to affordable safety resources such as fencing, gates, or construction labour. Similar to studies in Iran and Saudi Arabia, our findings indicate that low home safety and first aid knowledge among caregivers is compounded by heavy caregiving workloads, further constraining their capacity to implement safety improvements (35–37). These intersecting challenges highlight how limited confidence, restricted resources, and perceived barriers can significantly impede safety-enhancing behaviours.

Despite these barriers, strong family and community networks play a critical role in promoting safer home environments (13). Raising awareness among all family members, not only mothers, emerged as a key facilitator. Access to nearby health facilities also supports timely care, as shown in similar studies from Kenya (38). Educational interventions empowering mothers, such as an Egyptian training program that improved knowledge and practices on scald prevention, can effectively strengthen home safety (39). These supportive influences can also act as cues that encourage families to adopt safer practices. Overall, these enabling factors suggest that community-embedded approaches, supported by shared caregiving norms and strengthened family and social networks, may be particularly effective for reducing childhood injuries in rural Nepal.

### 4.4 Prevention and control practices for child injuries

Participants described prevention strategies that they viewed as both beneficial and achievable, emphasizing simple, practical measures to reduce home hazards, including constructing barriers with locally available materials and ensuring consistent supervision. This finding echoed from studies conducted in Uganda and Bangladesh (29, 40, 41). Suggested interventions included fencing, separating animal shelters, and keeping hazardous substances out of reach of children. These low-cost, locally sourced strategies demonstrate the value of community-driven, culturally appropriate approaches to injury prevention (42). Importantly, participants recognised that such changes were more feasible when they aligned with existing household routines and used materials readily available within the community. This highlights the importance of families feeling capable of taking manageable steps toward safer environments. Strengthening families’ confidence to implement these small, incremental modifications may therefore be a critical pathway to improving home safety in resource-constrained settings.

### 4.5 Design and delivery of IEC materials

A key contribution of this study was the exploration of IEC materials with effective formats and content for community-based injury prevention education. Participants valued materials that offered clear and actionable cues, preferring visual and low-literacy-friendly tools such as posters, flipcharts, and videos for their structured and accessible presentation of information. This finding aligns with studies in the UK and Austria emphasizing the effectiveness of visual and interactive communication (43, 44). Similarly, in several LMICs, the use of IEC materials for injury prevention has been recommended, including in the Philippines (45), Bangladesh (41), and Vietnam (46).

Group discussions emerged as an effective delivery method, promoting shared learning and community responsibility, consistent with participatory approaches in similar rural settings in Nepal (47). A study from Thailand found that both mothers and fathers valued the researcher-facilitated collaborative learning process, which provided opportunities to learn with, from, and about other parents (33). Evidence from Pakistan and Palestine supports combining in-home tutorials and printed materials to reduce household hazards, though group-based approaches may offer greater efficiency (45, 48).

However, challenges remain in ensuring the quality and sustainability of IEC interventions in Nepal. While FCHVs are trusted community educators, their limited technical training may constrain the depth of injury prevention education they can provide (49). Short training-of-trainers modules, supportive supervision, and job aids such as simple reference guides, checklists, or visual cue cards could enhance their capacity (50). Although professional facilitators, such as nurses, have demonstrated success elsewhere (51), FCHVs’ community presence and trustworthiness make them well suited for this role, particularly when equipped with tools that enhance their confidence to deliver clear, practical safety messages.

### 4.6 Strengths and limitations

This study captured diverse perspectives by engaging mothers of preschool children and a broad range of community stakeholders. Seven FGDs with 56 mothers from multiple ethnic groups and 11 KIIs with FCHVs, health-facility in-charges, a school nurse, and local officials enhanced the cultural relevance and contextual depth of the findings. The use of FGDs and KIIs, guided by the Health Belief Model, provided a strong conceptual foundation, while double coding, NVivo-supported analysis, and peer debriefing strengthened rigour and credibility.

Although this study was conducted in a single ward of one rural municipality, the findings are likely to be relevant to other hilly rural municipalities across Nepal. Given that hilly areas comprise around 68% of the country and nearly half of rural households (47.7%) are located in these regions, the results have potential applicability to approximately 266 similar municipalities. Nonetheless, the limited geographic scope, exclusion of fathers and other caregivers, potential social desirability bias, and reliance on self-reported data may have influenced the breadth and accuracy of responses. Seasonal factors, as data were collected in December, may also have affected the types of household injury risks identified.

### 4.7 Recommendations for practice, policy and future research

This study highlights the need for culturally and contextually relevant community-based strategies to address the risks of childhood home injuries in Nepal. Embedding injury prevention within existing community health structures, such as mothers’ groups and the routine work of FCHVs, offers a feasible and acceptable approach. Integrating age-appropriate injury prevention and home safety education into school curricula could further strengthen early safety awareness and injury prevention behaviours.

From a policy perspective, integrating home injury prevention into national child health and protection strategies would help ensure sustainability and wider impact. Local government leadership and support are essential to enhance community engagement and to allocate resources for preventive measures, such as safer home environments and educational materials.

There is a clear need for educational interventions that empower parents to recognize hazards, adopt safer practices, and create home environments that reduce the risk of childhood injuries. Such interventions should be designed to build parents’ confidence and practical skills, while also engaging wider family and community networks to share responsibility for child safety.

## 5 Conclusion

This study demonstrates community awareness of childhood injury risks and a willingness among parents to adopt safer practices when appropriately supported. The findings highlight the importance of culturally and contextually relevant, community-based strategies for home injury prevention in Nepal. A co-produced educational intervention, developed with parents, FCHVs, and local decision makers, and supported by a stakeholder advisory group, represents a promising and contextually grounded approach to enhance household safety. Embedding such interventions within trusted community health structures, such as mothers’ groups and the routine work of FCHVs, can maximize their relevance, acceptance, and long-term impact on reducing childhood injuries in rural Nepal and similar socio-economic settings.

## Data Availability

The original contributions presented in the study are included in the article/supplementary material, further inquiries can be directed to the corresponding author.
